# Polarization memory effect in the photoluminescence of nc-Si−SiO_x_ light-emitting structures

**DOI:** 10.1186/s11671-016-1496-4

**Published:** 2016-06-02

**Authors:** Katerina Michailovska, Ivan Indutnyi, Petro Shepeliavyi, Mykola Sopinskyy

**Affiliations:** V. Lashkaryov Institute of Semiconductor Physics Natl. Acad. of Sci. of Ukraine, 45 Prospect Nauky, 03028 Kyiv, Ukraine

**Keywords:** Silicon, Nanostructure, Photoluminescence, Polarization, Polarization memory effect, Quantum confinement effect, 78.67.Bf, 78.66.Db, 78.55.Mb

## Abstract

The polarization memory (PM) effect in the photoluminescence (PL) of the porous nc-Si−SiO_x_ light-emitting structures, containing nanoparticles of silicon (nc-Si) in the oxide matrix and passivated in a solution of hydrofluoric acid (HF), has been investigated. The studied nc-Si−SiO_x_ structures were produced by evaporation of Si monoxide (SiO) powder in vacuum and oblique deposition on Si wafer, and then the deposited silicon oxide (SiO_x_) films were annealed in the vacuum at 975 °C to grow nc-Si. It was found that the PM effect in the PL is observed only after passivation of nanostructures: during etching in HF solution, the initial symmetric nc-Si becomes asymmetric elongated. It was also found that in investigated nanostructures, there is a defined orientational dependence of the PL polarization degree (*ρ*) in the sample plane which correlates with the orientation of SiO_x_ nanocolumns, forming the structure of the porous layer. The increase of the *ρ* values in the long-wavelength spectral range with time of HF treatment can be associated with increasing of the anisotropy of large Si nanoparticles. The PM effect for this spectral interval can be described by the dielectric model. In the short-wavelength spectral range, the dependence of the *ρ* values agrees qualitatively with the quantum confinement effect.

## Background

Thin-film structures containing nanoparticles of silicon (nc-Si) embedded in the silicon oxide (SiO_x_) matrix attract the attention of many researchers, because of their promising applications in advanced electronic and optoelectronic devices [1–4]. The intensity and spectral range of the photoluminescence (PL) of these nanocomposites are determined mainly by the size and structure (amorphous or crystalline) of silicon nanoparticles, which in turn depends on the stoichiometry index of the oxide matrix and temperature of the forming annealing. Main PL characteristics (spectral, kinetics) and photoluminescence mechanism of nc-Si−SiO_x_ structures have been studied sufficiently, while the polarization properties of the PL, unlike for the porous silicon, have been sparsely investigated. In porous silicon, the polarization memory (PM) effect that is the correlation between polarization of excited light and polarization properties of PL was found, and its features and mechanism were studied [5–9]. But in the nc-Si−SiO_x_ structures, obtained using high-temperature annealing of non-stoichiometric SiO_x_ in inert atmosphere or vacuum, the PM effect was not observed. This may be explained by the fact that because of the isotropy of amorphous oxide, silicon nanoparticles formed during annealing are also isotropic. Thus, the effect of PM in these structures does not manifest itself.

In the previous works, it was shown [10, 11] that the treatment of these structures in solution or vapors of hydrofluoric acid can significantly increase the PL intensity and shift the PL peak position to short-wave region due to partial etching of nc-Si and passivation of their surface. Especially, effective etching and passivation takes place in porous nc-Si−SiO_x_ structures that are formed by oblique deposition of Si monoxide (SiO) in vacuum and the subsequent high-temperature annealing of the obtained SiO_x_ layer [12–14]. These layers have a porous columnar structure with oxide nanocolumns inclined at a certain angle to the sample surface. During high-temperature annealing of these films, the thermally stimulated formation of Si nanoinclusions occurs in a restricted volume of the SiO_x_ columns. Due to free space (cavities) between the oxide columns, the structures are more susceptible to chemical treatments, e.g., to etching in HF solution or vapor. Recently, it was shown for the first time [15] that after vapor fluorine-hydrogen treatment of such structures, the PM effect also manifests in them. It means that porous nc-Si−SiO_x_ structures can be used for fabrication of polarized light sources. Linearly polarized emission of these nanostructures may have potential uses as backlight for flat-panel displays [16] and as a biological labeling [17].

In this paper, we study the influence of HF aqueous solution processing time of porous nc-Si−SiO_x_ structures on their nc-Si morphology and the PL polarization properties.

## Methods

The investigated nc-Si−SiO_x_ light-emitting structures were produced by thermal evaporation of 99.9 % pure silicon monoxide SiO powder (Cerac Inc., Milwaukee, WI, USA) in vacuum ((1…2) × 10^–3^ Pa) onto polished nc-Si substrates. The substrates were arranged at the angle (*α*) of 60° relative to the normal to the substrate surface with the direction to the evaporator (oblique or glance angle deposition). The evaporation rate was monitored in situ by the KIT-1 quartz-crystal-oscillator monitor system. The deposited film thickness was measured, using MII-4 micro-interferometer, and amounted to 900 to 950 nm. The films were annealed in vacuum for 15 min at the temperature of 975 °C. Passivation of the nc-Si−SiO_x_ structures, obtained in this manner, was carried in an aqueous solution of the HF. The effect of treatment on the PL spectra was studied by varying the time of treatment at a certain temperature (20 °C) of the solution and at a certain concentration (0.5 wt% HF).

The PL spectra were excited using linearly polarized emission of a semiconductor laser at the 415 nm wavelength. Polarization of the exciting light was rotated by an achromatic half-wave plate and cleaned by a linear polarizer. The orientation of the polarization vector of the emitted light was defined by a sheet polarizer (analyzer) placed in the detection path. PL was excited and detected in nearly normal to the sample surface. PL spectra were measured at room temperature within the wavelength range from 550 to 850 nm. These spectra were normalized to the spectral sensitivity of the experimental system and were corrected with respect to the polarization dependent response of the measurement system.

## Results and discussion

The structure of obliquely deposited SiO_x_ films was studied by the SEM apparatus (ZEISS EVO 50XVP, Oberkochen, Germany) in previous papers [10, 12]. The film structure presents well-defined columns characterized by a certain orientation of growth; the column diameter varies in the 10–100 nm range. The dimensions of the columns, their orientation, and the porosity (the relative volume of pores) of the films depend on the angle of deposition. At the angle *α* = 60°, the porosity equals to 34 % and the inclination angle of the formed oxide nanocolumns relative to the normal to the sample surface is 26…29° [12, 18]. The porosity of the films and the dimensions and inclination of the columns remained unchanged on annealing [14]. After annealing, the obtained nc-Si−SiO_x_ structures show weak PL. The PM effect in the spectra of PL is not observed, despite the fact that they possess biaxial optical anisotropy [18]. But the HF treatment of the samples is accompanied by a gradual change in the emission spectra and their polarization properties.

**Fig. 1:**
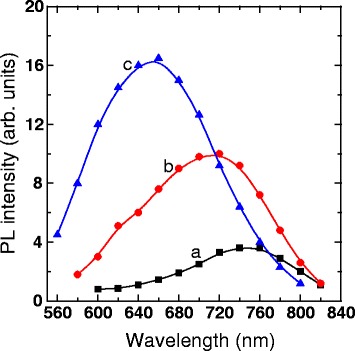
PL spectra of porous nc-Si−SiO_x_ sample annealed in vacuum, then treated in 0.5 % HF solution for 5 (*a*), 10 (*b*), and 17 (*c*) min. The spectra were measured for parallel orientation of the analyzer to polarization of the exciting radiation, and polarization of the exciting light is oriented parallel to the projection of the inclined SiO_x_ nanocolumns on the sample plane

Figure 1 shows the PL spectra of nc-Si−SiO_x_ sample deposited in vacuum at the angle *α* = 60°, annealed at 975 °C in vacuum and then etched in 0.5 wt% HF solution for 5 (a), 10 (b), and 17 (c) min. These results correspond to the orientation of the analyzer, which selects polarization of PL parallel to polarization of exciting radiation. The sample is oriented so that the polarization of the exciting light is parallel to the projection of the inclined SiO_x_ nanocolumns on the sample plane. The nc-Si−SiO_x_ structure exhibits the broad PL band within the 550 to 820 nm wavelength range which can be attributed to exciton recombination in nc-Si [19]. These spectra have similar line shapes; however, the positions of their emission peak and intensities are largely different. By increasing the etching time, we observe a gradual shift of the position of emission peak to shorter wavelengths and an increase in the PL intensity. The blue shift of PL band in HF-treated samples can be attributed to the selective-etching-induced decrease in the Si nanoparticle dimensions [13]. In the porous films studied here, HF penetrates deep into the film and dissolves SiO_2_ at the surface of the oxide columns, thus stripping nc-Si. After HF treatment, the nc-Si again forms a thin native oxidized layer on the surface when exposed to the atmospheric oxygen. Because of oxidation of the nc-Si surface, the dimensions of the initial nc-Si core are reduced, resulting in the blue shift of the emission spectrum. Significant enhancement in the PL intensity after HF treatment is related to the passivation of Si dangling bonds (non-radiative recombination trap states) by hydrogen and oxygen [11, 14].

As has been shown in our previous paper [15], etching in HF vapor can change also the shape of symmetric Si nanoparticles to the elongated anisotropic one, similar to that in porous silicon, and leads to the PM effect. Indeed, in such samples, the intensity of PL polarized in parallel to polarization of excitation is higher than the PL component polarized in the perpendicular direction, i.e., in the investigated samples, the PM effect is really observed. This effect can be illustrated by the degree of linear polarization of the PL, which is known to be defined with the expression:

1 $$ \rho =\frac{I_{II}-{I}_{\perp }}{I_{II}+{I}_{\perp }}, $$

where *I*_*II*_ and *I*_┴_ are the intensities of the photoluminescence with polarization parallel and perpendicular to that of the excitation light, respectively.

**Fig. 2:**
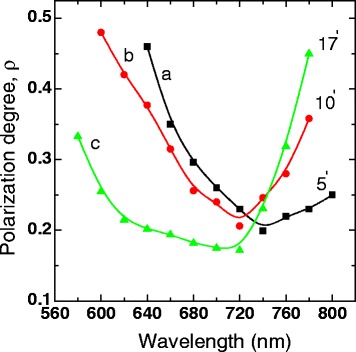
Spectra of the polarization degree for porous of nc-Si−SiO_x_ sample, in which PL spectra are shown in Fig. 1. The numbers near the curves indicate the time of treatment in HF solution in minutes

The spectral dependences of the *ρ* for the same samples as in Fig. 1 are shown in Fig. 2. For all investigated samples, there is an observed non-monotonous variation of the degree of linear polarization over the whole spectra range. In the long-wave region of emission (730–800 nm), which corresponds to larger sizes of Si nanoparticles, the *ρ* value increases with the etching time and grows into long-wavelength part of the spectrum. For the sample etched 5 min (a), the *ρ* value grows more smoothly in comparison with the samples etched 10 (b) and 17 (c) min. In the ranges near the PL maxima, the *ρ* have the minimal values; then, it increases also by moving toward higher emitted energy. But unlike the long-wave region of PL, the increase of *ρ* value in short-wave region occurs more sharply for the sample etched 5 min (a), i.e., for smaller etching time.

These results are somewhat different from the dependencies that were obtained in our previous works [15, 20] for porous nc-Si−SiO_x_ samples etched in HF vapor. In the vapor-treated samples, the *ρ* slightly decreases with increasing wavelength in the long-wavelength spectral region, similar to how it happens in porous silicon [5–9]. This difference in observed behavior of *ρ* in the long-wavelength spectral region may be connected with the difference in the etching mechanism of the porous structure in the liquid or vapor phase.

**Fig. 3:**
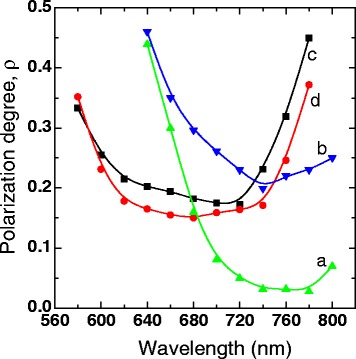
Spectra of the polarization degree for porous of nc-Si−SiO_x_ sample, which were treated in HF solution for 5 (*a*, *b*) and 17 (*c*, *d*) min. The polarization of the exciting light was oriented parallel to the projection of the inclined SiO_x_ nanocolumns on the sample plane (*b*, *c*) and in perpendicular direction (*a*, *d*)

Figure 3 shows *ρ* spectra for the samples which were HF-treated for 5 (a, b) and 17 (c, d) min. The polarization of the exciting radiation is oriented parallel to the projection of the inclined SiO_x_ nanocolumns on the sample plane (b, c) and in perpendicular direction (a, d). It can be seen that for the sample that was HF-treated for 5 min, the PM effect is pronounced more efficiently for parallel orientation of the excitation polarization and the nanocolumns’ projection than for perpendicular orientation. At the energy of PL maximum, the *ρ* values are equal to 0.22 (b) and 0.03 (a). This means that the anisotropic elongated silicon nanoparticles have preferred orientation in the film and the projection of this orientation on the plane of the sample coincides with the direction of projection of the SiO_x_ nanocolumns. It can be assumed that the orientation of the longer axes in most of nc-Si particles coincides with the orientation of SiO_x_ nanocolumns. This assumption is consistent with the conditions of etching of the porous matrix: dissolution occurs starting from the surface of the SiO_x_ nanocolumns; first, the side surface of nc-Si is dissolved, since it is closer to the column surfaces, which leads to elongation of nanoparticles along the axis of the column. Obtained results are consistent with earlier studied angular dependencies of the PL intensity in the porous nc-Si−SiO_x_ structures passivated in HF vapor which indicate the well-defined orientation dependence of *ρ* in the sample plane [20]. This result is similar to that observed in porous silicon formed by electrochemical etching of Si with orientation [100] in the presence of linearly polarized light illumination [6], which indicated the existence of PM anisotropy in the plane of the sample.

For the sample that was treated for 17 min, the PM effect is almost the same for both orientations of the excitation polarization relative to the sample, and the ρ values at the PL maximum are equal to 0.20 (c) and 0.16 (d), for parallel and perpendicular orientation of the excitation polarization and SiO_x_ nanocolumns projection, respectively. As has been shown previously by electron microscopic studies [14], long-duration etching of our samples leads to a reduction in their thickness, etching of the nanocolumns, and the formation of isotropic disordered structure. As in the continuous (solid) nc-Si−SiO_x_ structures [15], in the plane of this sample, there are no preferential orientations of anisotropic silicon nanoparticles.

There were several mechanisms proposed that are responsible for the PM effect in silicon nanostructures, where the two main ones are the following: modification of energy spectrum and optical matrix elements by size quantization of carriers [21–23] and dielectric confinement of optical electric field caused by the difference in the dielectric constants of the nanoparticle and the environment [5, 24]. In [21], the linear character of PL polarization in porous Si is studied experimentally and theoretically using a quantum cylindrical model in the framework of the effective-mass theory. From the experimental and theoretical results, it is concluded that PL polarization anisotropy of elongated particles associated with the structure of the valence band due to quantum confinement in two directions—parallel and perpendicular to the longer axis of the particle.

However, there exists the more common explanation of the PM effect—within the dielectric model in which porous silicon is considered as a composite that includes elongated and flattened silicon nanocrystals [5–7]. The probability of optical absorptions and emission is proportional to the square of the electric field inside the nc-Si, and therefore, nanocrystals with their longest dimensions aligned along the polarization direction of exciting light will preferentially absorb and emit photons. Then, the PM is the result of selective excitation that part of the non-spherical silicon nanoparticles whose longer axis is parallel to polarization of exciting radiation [5, 8, 24]. Polarization properties of individual silicon nanorods with diameter around 5 nm, embedded in SiO_2_ and oriented parallel to the Si substrate, are studied using an optical micro-spectroscopy setup [25]. Experimental results are compared with available theoretical models leading to the conclusion that the high polarization degree is mostly due to surface charges (dielectric confinement) with smaller contribution of quantum confinement effects. Both interpretations as on the basis of quantum size effects and within the dielectric model associate the PM effect with asymmetric, elongated nanoparticles that emit PL.

On the basis of these models, it is possible to explain the features of our results. The broad PL bands in our samples (Fig. 1) are the superposition of radiation from nc-Si with different sizes; the smaller particles correspond to a more short-wave radiation. The position of PL maximum is determined by the maximum in the size distribution of nanoparticles.

The increase of the *ρ* values in the long-wave spectral range and with time of HF treatment (Figs. 2 and 3) can be associated with the increase of the asymmetry of large Si nanoparticles, for example, with the increase of eccentricity of elongated ellipsoidal nc-Si. The PM effect for this spectral interval is most likely associated with the dielectric model. Using dielectric model, we estimated the asymmetry of nanoparticles emitting near PL maximum for the sample treated in HF solution for 5 min. We assume that the nc-Si are elongated ellipsoids of rotation with the semi-axes *a*, *b*, *c* (*a = b*, *c > a*) and that the value of the angles between the long axis of ellipsoids and the normal to the sample surface is equal to the columns inclination angle value. Using formulas from the paper [5] and the values of optical dielectric constants inside (*ε*_*i*_ = 15 [26]) and outside nc-Si (*ε*_o_ = 1.5), we found that the *ρ* values are close to the experimental ones (Fig. 3, curves a, b) if the ellipsoids have *a*/*c* ≈ 0.36. Since the *ρ* values slightly increase toward longer wavelengths, the eccentricity of larger nanoparticles somewhat increases too.

The increase of the *ρ* values in the long-wave spectral range with time of HF treatment (Fig. 2(b, c)) can be associated with the increase of the asymmetry of large Si nanoparticles. With further increase of HF treatment time, along with an increase in nc-Si asymmetry, their preferred orientation are also destroyed, as can be seen in Fig. 3(c, d). We interpreted the experimental data for the samples that were HF-treated for 17 min assuming that these samples can be considered as an ensemble of randomly oriented elongated nc-Si, embedded in an effective dielectric medium. The *ρ* value for such ensembles with randomly orientation of ellipsoids axes was calculated in [27, 28]:

2 $$ \rho =\frac{{\left(1-{k}^2\right)}^2}{7{k}^4-6{k}^2+2}. $$

The *k* value is related to the depolarizing factor *n* of the ellipsoid by

3 $$ k=\frac{2+2\left({\varepsilon}_i/{\varepsilon}_0-1\right)n}{2+\left({\varepsilon}_i/{\varepsilon}_0-1\right)\left(1-n\right)}, $$

where the depolarizing factor *n* is calculated according to the expression [29]:

4 $$ n=\frac{{\left(a/c\right)}^2}{{\left[1-{\left(a/c\right)}^2\right]}^{3/2}}\left[ Arc \tanh \sqrt{1-{\left(a/c\right)}^2}-\sqrt{1-{\left(a/c\right)}^2}\right]. $$

Using formula (1)–(4) and the same values of the optical dielectric constants, it was determined that, near PL maximum, the *ρ* value is equal to 0.18 if the elongated ellipsoids have *a*/*c* ≈ 0.4. As seen, this calculated *ρ* value is close to experimental ones (≈0.19). However, in a more long-wave spectral range, the *ρ* increases sharply enough with the increase of the wavelength (Fig. 3, curves c, d). In this spectral range the ρ values, calculated by the formulas (1–4), are approximate to the experimentally obtained ones if we assume that, in consequence of HF treatment, emitting nc-Si ellipsoids of the larger sizes have *a*/*c* ≈ (0.1–0.2), i.e., their eccentricity is essentially increased. As shown in [30], in a random system of nanowires, excited by polarized light, the maximal *ρ* value is equal to 0.5.

We see also that the increase in time of HF treatment in the investigated interval of times has a considerably smaller effect on the asymmetry of Si nanoparticles which are responsible for the emission in the range near the PL maxima. With the further decrease of the nc-Si size, i.e., in the short-wave region of the PL band, the increase of *ρ* with the decrease of wavelength, as in porous silicon, agrees qualitatively with the quantum-confined nanostructure model in which the degree of polarization increases with decreasing nanostructure size [21, 22].

## Conclusions

We investigated the polarization memory effect in the PL of nc-Si−SiO_x_ light-emitting nanocomposites in which the silicon nanoparticles are embedded into the optically anisotropic SiO_x_ matrix possessing a porous column-like structure. It was found that the degree of PL linear polarization depends on the time of the samples treatment in HF water solution. During etching in HF, the nanoparticles with initial spherical shape become asymmetric elongated, preferably in a direction along oxide nanocolumns. This results in experimentally observed well-defined orientation dependence of the *ρ* value in the sample plane. But long-duration HF treatment of our samples leads to etching of the nanocolumns and the formation of isotropic disordered structure.

We have concluded that observed spectral dependence of the *ρ* value in nc-Si−SiO_x_ structures in long-wavelength region can be explained on the basis of classical surface charge (dielectric) model. In short-wave region of emission spectra, it is necessary to take into consideration the contribution of quantum confined model in which the degree of polarization increases with decreasing nanostructure size.

## Abbreviations

HF, hydrofluoric acid; KIT-1, quartz-crystal-oscillator monitor system; MII-4, micro-interferometer; nc-Si, silicon nanoparticles; PL, photoluminescence; PM, polarization memory; SEM, scanning electron microscopy
